# Merging Nitrogen Management and Renewable Energy Needs

**DOI:** 10.1100/tsw.2001.370

**Published:** 2001-11-22

**Authors:** Elizabeth Wilson, Pippa J. Chapman, Adrian McDonald

**Affiliations:** School of Geography, University of Leeds, UK

**Keywords:** ARBRE, nitrogen, renewable energy, sewage sludge, short rotation coppice, Urban Waste Water Treatment Directive

## Abstract

The ARBRE (ARable Biomass Renewable Energy) project, the first large-scale wood-fueled electricity generating plant in the U.K., represents a significant development in realising British and European policy objectives on renewable energy. The plant is fueled by a mix of wood from short rotation coppice (SRC) and forest residues. Where feasible, composted/conditioned sewage sludge is applied to coppice sites to increase yields and improve soil structure. In the Yorkshire Water region, typical total N:P:K composition of composted/conditioned sludge is 2.9:3.8:0.3, respectively. Sludge application is calculated on the basis of total nitrogen (N) content to achieve 750 kg N ha, for 3 years’ requirement. Willow coppice forms a dense, widely spaced, root network, which, with its long growing season, makes it an effective user of nutrients. This, in combination with willow’s use as a nonfood, nonfodder crop, makes it an attractive route for the recycling of sewage sludge in the absence of sea disposal, banned under the EC Urban Waste Water Treatment Directive (UWWTD). Further work is required on the nutritional requirements of SRC in order to understand better the quantities of sludge that can be applied to SRC without having a detrimental impact on the environment. This paper suggests the source of N rerouting under the UWWTD and suggests the likely expansion of SRC as an alternative recycling pathway.

## INTRODUCTION

In response to the threat of climate change, the developed countries agreed at Kyoto in 1997, to legally binding targets to reduce their emissions of the six main greenhouse gases. The EU agreed to an 8% reduction relative to 1990 levels, over the period 2008–2012, and within this, the U.K. agreed to cut its emissions by 12.5%. Renewable sources of electricity form a key component in the drive to reduce greenhouse gas emissions. At the end of 1999, renewable energy sources represented 2.8% of total electricity generated in the U.K.[1]. In February 2000 the government set a domestic target to increase the contribution of electricity supplied from renewables to 5% by the end of 2003, and 10% in 2010[2].

The ARBRE (ARable Biomass Renewable Energy) project, which was commissioned in April 2001, is the first large-scale, wood-fuelled, electricity generating plant in the U.K. and represents a significant development in realising U.K. and European policy objectives on renewable energy. The plant, located at Eggborough, near Selby, in North Yorkshire, uses gasification technology to supply 8 MW of electricity to the local grid. The project has received financial support through inclusion in the third tranche of the U.K.'s Non Fossil Fuel Obligation (NFFO)

and also in the EU's THERMIE programme of support for the demonstration and dissemination of new, clean, and efficient energy technologies. The wood fuel requirement of the plant is approximately 43,000 oven dry tonnes (odt) year$^{-1}$, which will come from a mix of willow short rotation coppice (SRC) and forest residues. SRC for the ARBRE project is planted at densities of 15,000 willow cuttings per hectare in double rows. Following the first year's growth, the coppice is cut back to ground level to encourage multiple stems, and after cutback the crop is harvested on a 3-year cycle for 15 years. To date, 1300 ha of SRC have been planted for the ARBRE project within a 60-km radius of the plant. Although the initial goal was to supply 80% of the fuel from SRC, this goal is now nearer 40%, as forest residues are cheaper, although, as the cost of SRC falls, it is hoped that a larger proportion of fuel will come from this source in the future.

At present very few ARBRE SRC sites have been harvested, and thus it is uncertain what yields can be expected from the sites. However, figures quoted in the literature would suggest that yields of SRC are typically in the range of 9 to 12 odt ha$^{-1}$ year$^{-1}$[3,4]. If a yield of 10 odt ha$^{-1}$ year$^{-1}$ and a N content in the stemwood of 0.3%[5] is assumed, this represents an export of 30 kg N ha$^{-1}$ year$^{-1}$ in the harvested stem wood. A portion of the N requirement of the growing crop will be supplied from leaf litter recycling, N fixation, and atmospheric N deposition, but it may also prove beneficial to apply fertiliser to the SRC crop in order to replace the nutrients removed at harvest. Fertilisation of SRC with inorganic fertilisers is generally uneconomic, but use of sewage sludge as an organic fertiliser can be beneficial both in terms of improved site fertility and through providing a suitable outlet for the recycling of sewage sludge. This paper will discuss the use of SRC as an outlet for the recycling of sewage sludge in the U.K.

## DISPOSAL OF SEWAGE SLUDGE IN THE U.K.

The most recent survey of sewage sludge production in the U.K.[6] reports results for the year 1996/7, during which 1,116,000 tonnes dry solids (tds) of sewage sludge was produced in the U.K. The principal disposal outlet for this sludge was recycling to agricultural land, accounting for 47% of sludge production, followed by sea disposal (25%), with smaller amounts going to incineration (8%), landfill (8%), land reclamation (6%), and to dedicated sites (3%) (Table 1). However, the picture painted by the 1996/7 survey must be regarded, to a certain extent, as transitional. Over the last decade, tightening legislative controls and increasing pressure from the British Retail Consortium (BRC) have had a significant impact on the various sludge disposal outlets. The EC Urban Waste Water Treatment Directive (UWWTD), which was adopted in 1991, required all member states to cease disposing of sewage sludge to sea by the end of 1998. In addition, the Directive requires sewage discharges to the marine environment, which were previously untreated, to receive treatment and also requires other, partially treated sewage to receive a higher level of treatment. It is projected that this requirement will result in a 31% increase in the volume of sewage sludge produced between 1996/7 and 2005/6[6]. It is expected that the additional sludge from increased production and phasing out of sea disposal will be recycled to agriculture, incinerated, or incinerated with energy recovery (Table 1).

In addition to the pressure placed on land-based sewage disposal routes by the requirements of the UWWTD, the agricultural outlet has been subject to considerable scrutiny in recent years by food retailers, as represented by the BRC. The BRC expressed concern relating to disease risks associated with sludge application (both treated and untreated) to land and the effects

**TABLE 1**
**Disposal Outlets for Sewage Sludge in the U.K. in 1990/1, 1996/7, and Projected Figures for 2005/6**

| | 1990/1* | | 1996/7† | | 2005/6† | |
|---|---|---|---|---|---|---|
| | tds year$^{-1}$ | %++ | tds year$^{-1}$ | %++ | tds year$^{-1}$ | %++ |
| Agriculture | 465 000 | 42 | 520 000 | 47 | 732 000 | 50 |
| Dedicated site | 25 000 | 2 | 39 000 | 3 | 38 000 | 3 |
| Incineration | 77 000 | 7 | 91 000 | 8 | 315 000 | 21 |
| Sea Disposal | 334 000 | 30 | 280 000 | 25 | - | - |
| Landfill | 88 000 | 8 | 91 000 | 8 | 56 000 | 4 |
| Land reclamation | - | - | 64 000 | 6 | 74 000 | 5 |
| Forestry | - | - | 1000 | <1 | 3 000 | <1 |
| Horticultural compost | - | - | 13 000 | 1 | 2 000 | <1 |
| Within curtilage | 50 000 | 5 | 15 000 | 1 | - | - |
| Other‡ | 68 000 | 6 | 1000 | <1 | 247 000 | 17 |
| Total | 1 107 000 | 100 | 1 116 000 | 100 | 1 467 000 | 100 |

* = Source: Department of the Environment [10].
† = Source: Gendebien et al.[6].
‡ Others: 1990/1 — more general category described as 'Beneficial'; 1996/7 — Uncertain; 2005/6 — Energy recovery through gasification and production of aggregates.
++ = % of total volume of sewage sludge.

on the food chain as a whole. As a result of agreement between the U.K. water industry and the BRC in 1998 regarding the safe application of sewage sludge to agricultural land, 'The Safe Sludge Matrix'[7] was published. It specifies target dates by which applications of untreated sludge must be phased out and those crops where the application of conventionally treated (to ensure 99% reduction in pathogens) and enhanced-treated (to ensure 99.9999% reduction in pathogens) sludge is considered acceptable. The requirements of the Matrix are to be included in the revised Sludge (Use in Agriculture) Regulations 2001, to be published later this year. The revised Regulations will go beyond the requirements of the 1986 EC Directive on the protection of the environment and, in particular, the soil, when sewage sludge is used in agriculture[8], which is what the original 1986 Sludge (Use in Agriculture) Regulations[9] were based on.

## DEVELOPMENT OF SRC AS AN OUTLET FOR THE RECYCLING OF SEWAGE SLUDGE

The increased constraints on agricultural disposal routes have prompted water utilities to look for new and more secure disposal outlets for sludge. For example, in the early 1990s Yorkshire Water identified SRC as a possible outlet for the recycling of sewage sludge. It was recognized that the development of a renewable energy project, which utilized wood from SRC, would serve the duel purpose of providing a source of renewable energy and an alternative outlet for the disposal of sewage sludge. First Renewables Ltd., a sister company of Yorkshire Water, established ARBRE Energy with TPS Termiska Processer of Sweden to develop the ARBRE project. The ability to apply sludge to ARBRE SRC sites is potentially of great significance to Yorkshire Water, in conjunction with other recycling routes. Where feasible, Yorkshire Water applies composted/conditioned sewage sludge to the SRC sites. Yorkshire Water began recycling significant quantities of sewage sludge to SRC in 2000, when approximately 8000 tonnes of sewage sludge was spread on SRC. In 2001 this figure has risen to approximately 11,800 tonnes. It is thought that the application of sewage sludge to SRC may increase to 20,000 tonnes/year in the next few years, but is unlikely to increase further, due to restrictions on the distance Yorkshire Water is prepared to transport sewage sludge to SRC sites[11].

Typical total N:P:K composition of composted/conditioned sewage sludge in the Yorkshire Water region is 2.9:3.8:0.3, respectively. Provided that the levels of heavy metals in the soil do not exceed 80% of the legislative levels laid out in the Sludge (Use in Agriculture) Regulations 1989[9], sludge is applied, according to total N content, at a maximum rate of 250 kg N $ha^{-1}$ $year^{-1}$. The availability of N from sewage sludges applied to land, nationally in various climatic and land-type conditions, is currently under long-term investigation, and initial indications from this work indicate that mineralisation of applied N varies between 5 and 20%/year[11]. The 250 kg N $ha^{-1}$ $year^{-1}$ rate of application is based on the maximum level recommended in the Code of Good Agricultural Practice for the Protection of Water (1998)[12] for total N applied to agricultural land in organic wastes. Sludge can be applied to the SRC prior to planting, after cutback, and after each harvest. SRC is grown on 3-year cycles, and where sludge is applied after harvest, it is applied at a rate of 750 kg N $ha^{-1}$ for 3 years growth, i.e., 250 kg N $ha^{-1}$ $year^{-1}$. However, during establishment of the SRC crop it may be desirable to apply sludge to the site prior to planting, in order to give the crop a 'head start' in the initial competition with weed species. Where this is the case, sludge is applied at a rate of 375 kg N $ha^{-1}$ prior to planting, and an additional 375 kg N $ha^{-1}$ is applied after cutback. This again gives a total of 750 kg N $ha^{-1}$, although, in the instance of the first rotation, this is spread over 4, not 3 years, giving a rate of application slightly below 250 kg N $ha^{-1}$ $year^{-1}$.

The use of SRC as an outlet for the recycling of sewage sludge has a number of advantages compared to conventional disposal routes:

1. The use of SRC as a nonfood, nonfodder crop means that there is no theoretical risk of pathogen exposure via the food chain. Consequently, the application of sewage sludge to SRC and other industrial crops has not come under the same sort of pressure from the BRC as application to food crops, and this outlet can generally be regarded as more secure.
2. From the perspective of the water utilities, disposal to SRC is attractive, as this route is potentially secured for 16 years (the crop is grown on 3-year cycles for 15 years, plus the initial cutback year) and offers a regular outlet. In addition, compared to conventional agriculture, up to three times the volume of sewage sludge is spread in one application (equivalent to 750 kg N $ha^{-1}$ in one application to SRC for 3 years growth, compared to up to 250 kg N $ha^{-1}$ per year for conventional agricultural disposal routes).
3. Compared to conventional forestry, SRC has more windows of opportunity for the application of sewage sludge: preplanting, postcutback, and postharvest. In addition, the frequent removal of nutrients in the harvested stems supports the more frequent applications of sewage sludge in order to replace nutrients removed at harvest.
4. Willow coppice forms a dense, widely spread root network and has been found by both laboratory and field experiments to be highly efficient in taking up available nutrients[13,14, 15]. Thus, the application of sewage sludge to SRC should ensure that the quality of groundwater should not be affected[13].
5. SRC for energy production is based on the concept of sustainability, and where the quality of sludge allows application to agricultural land, this is usually regarded as the Best Practicable Environmental Option (BPEO)[16] and is more environmentally acceptable than the alternative options of incineration or landfill. In addition, where sewage sludge is applied to SRC and replaces the use of inorganic fertiliser, it conserves the energy used to produce the inorganic fertiliser.

## FURTHER RESEARCH NEEDED ON THE FERTILISATION OF SRC WITH SEWAGE SLUDGE

SRC is a fairly new cropping system, and there is little information on yield response of SRC to sewage sludge or on the effects of fertilisation on site nutrient dynamics. SRC growers need research to ascertain the optimum amount and timings of application of sewage sludge that should be applied in order to obtain the maximum yield. From the perspective of the water utilities, research is required to quantify the maximum volume of sewage sludge that can be applied to SRC without unacceptable loss of nutrients through leaching to surface and groundwaters. At present, sewage sludge is applied to SRC at the maximum rate recommended in the Code of Good Agricultural Practice for the Protection of Water[12]. This rate of 250 kg N $ha^{-1}$ $year^{-1}$ was developed with regard to arable crops. Yorkshire Water applies sewage sludge at this rate, as it is the only guideline available, but it would be more appropriate for the industry to develop recommended rates of application specific to SRC.

Future research should also consider the real limiting factors on the growth of SRC — the two principal factors being nutrient and water availability. SRC is known to have a high water demand[17,18], and this should be taken into consideration when recommending fertilisation rates. In addition, recommended fertilisation rates should also consider the different types of sewage sludge that can be applied to SRC. Under the 1998 agreement between the U.K. Water industry and the BRC, applications of untreated sludge are permitted to industrial crops until 2005, whereupon only applications of conventionally and enhanced-treated sludge are allowed[19]. There are a number of different treatment options for sewage sludge, all of which will have differing effects on the properties of the sewage sludge. Of particular importance for SRC are treatment processes that affect nutrient availability, dry solids content, and water holding capacity of the sludge.

## CONCLUSIONS

This paper has described an innovative way in which humans are rerouting the flows of N for the benefit of the environment. A portion of the sewage sludge, which prior to 1998 was dumped at sea, is now being recycled to SRC. By recycling sewage sludge to SRC, the nutritional and soil conditioning properties of the sludge are utilized, and it is treated more as a resource rather than just a waste product. In turn, the SRC is used to fuel a renewable energy plant that generates electricity with minimal emissions of greenhouse gases compared to conventional power stations.

At present, SRC is of significance as a recycling outlet for sewage sludge in the Yorkshire Water region and surrounding regions to the south and east. However, First Renewables Ltd. hopes to build up to ten more of the ARBRE type plants across the U.K., each four to five times bigger than the existing plant, and each requiring up to 7000 ha of biomass crops as fuel, along with other fuel sources such as forestry. Thus, it would appear that there is considerable scope for SRC to increase in significance in the future as an outlet for the recycling of sewage sludge.

## ACKNOWLEDGEMENTS

This research is funded jointly by Yorkshire Water and First Renewables Ltd., and we would like to thank Keith Pitcher of First Renewables Ltd. and Jon Brigg of Yorkshire Water for their comments and suggestions relating to this paper.

| | | |
|---|---|---|
| Received: | July | 27, 2001 |
| Revised: | November | 5, 2001 |
| Accepted: | November | 9, 2001 |
| Published: | November | 22, 2001 |

## BIOSKETCH

**Elizabeth Wilson** is a research postgraduate at the School of Geography, University of Leeds, U.K. She has a first class degree in Geography, also from the University of Leeds. Her research is investigating the effect of applications of sewage sludge to short rotation coppice site nutrient dynamics, with a particular emphasis on nitrogen cycling. Her research is sponsored jointly by First Renewables Ltd. and Yorkshire Water.

**Adrian McDonald** is Professor of Environmental Management at the University of Leeds, U.K. Previously he was Dean of the Earth and Environment Faculty and Associate Professor at the University of Guelph, Ontario. He has a first class degree in Resource Management from the University of Edinburgh and a Ph.D. from the same institution. He has 25 years experience of applied research in the water environment. His interests cover catchment and river management, demand estimation and forecasting and biofuel based renewable energy.

**Pippa Chapman** is a lecturer in physical geography at the University of Leeds, UK. Her research interests are concerned with the impacts of atmospheric deposition and land use management on nutrient cycling, particularly nitrogen and phosphorus, in soil and surface waters.
